# Sexual functioning in women with functional hypothalamic amenorrhea: exploring the relevance of an underlying polycystic ovary syndrome (PCOS)-phenotype

**DOI:** 10.1007/s40618-023-02021-7

**Published:** 2023-02-03

**Authors:** F. Barbagallo, G. Pedrielli, D. Bosoni, L. Tiranini, L. Cucinella, A. E. Calogero, F. Facchinetti, R. E. Nappi

**Affiliations:** 1 Department of Clinical and Experimental Medicine, University of Catania, Policlinico “G. Rodolico”, via S. Sofia 78, 95123 Catania, Italy; 2 Department of Medical and Surgical Sciences for Mother, Child and Adult, University of Modena and Reggio Emilia, Azienda Ospedaliero Universitaria Policlinico, Modena, Italy; 3 Department of Clinical, Surgical, Diagnostic and Pediatric Sciences, University of Pavia, Pavia, Italy; 4 Research Center for Reproductive Medicine, Gynecological Endocrinology and Menopause, IRCCS San Matteo Foundation, Pavia, Italy

**Keywords:** Functional hypothalamic amenorrhea, Polycystic ovary syndrome, Sexual function, Mood, Androgens

## Abstract

**Purpose:**

To study sexual function and distress in women with functional hypothalamic amenorrhea (FHA) compared to women with FHA and an underlying polycystic ovary syndrome (PCOS)-phenotype, considering also their psychometric variables. As a secondary aim, we explored the relationship between sexual functioning and hormonal milieu in these women.

**Methods:**

This is a retrospective cross-sectional study conducted on 36 women with typical FHA and 43 women with FHA + PCOS-phenotype. The following validated psychometric questionnaires were administered: Female Sexual Functional Index (FSFI), Female Sexual Distress Scale-Revised (FSDS-R), Body Attitude Test (BAT), Bulimia Investigation Test (BITE), State Anxiety Inventory (STAI), Beck Depression Inventory (BDI), Multidimensional Perfectionism Scale (MPS). Available hormones to formulate FHA diagnosis in the standard routine were considered.

**Results:**

Women with typical FHA reported a significantly lower FSFI total score than women with FHA + PCOS-phenotype (95% CI for median 16–21.3 vs. 21.1–24.1, *p* = 0.002), whereas the FSDS-R score was similar in the two groups (95% CI for median 6–16 vs. 6–16.3). No statistically significant differences were evident in body attitude, state and trait anxiety, depression, bulimic risk, and perfectionism between the two groups, confirming the two FHA groups were superimposable from a psychometric perspective. State anxiety correlated negatively with the FSFI total score in both typical FHA (rho: − 0.33, *p* = 0.05) and FHA + PCOS-phenotype (rho: − 0.40, *p* = 0.009). In the entire study population, a positive correlation was found between luteinizing hormone, androstenedione, and 17ß-estradiol and the total FSFI score (rho: 0.28, *p* = 0.01; rho: 0.27, *p* = 0.01, rho: 0.27, *p* = 0.01, respectively).

**Conclusion:**

Women with FHA showed a very high rate of sexual symptoms as part of their condition, but those with a typical diagnosis displayed a more severe sexual impairment as compared with the FHA + PCOS-phenotype, in spite of a similar psychometric profile. Sexual distress was equally present in both groups (approximately 4 out of 10 women). Further studies should be designed to investigate the potential role of sex hormones, mainly LH-driven androstenedione, in influencing women’s sexual functioning.

**Supplementary Information:**

The online version contains supplementary material available at 10.1007/s40618-023-02021-7.

## Introduction

A bidirectional relationship exists between stress response and reproductive function, ultimately affecting also sexual behavior in both sexes [1]. Functional hypothalamic amenorrhea (FHA) is the term used to describe the lack of menstruation not related to organic causes but resulting from various stressors. It is typically associated with three main causes: weight loss, excessive physical exercise, and psychological stress, which can occur alone or, more often, in combination [2]. FHA is responsible for 20–35% of secondary cases of amenorrhea, and about 3% of women with primary amenorrhea [3].

The hallmark of FHA diagnosis is hypogonadotropic hypogonadism related to the suppression of the pulsatile release of hypothalamic gonadotropin-releasing hormone (GnRH) which in turn impairs the pulsatile release of luteinizing hormone (LH) with reduced secretion of 17ß-estradiol (E_2_) by the ovary [4]. Numerous hormonal and non-hormonal factors influence the pulsatile secretion of GnRH and come into play in the pathophysiology of FHA, which is rather complex and still not completely understood [2, 4]. Chronic stress is associated with a constellation of neuroendocrine adaptive responses [4, 5] which may influence sexual behavior throughout several common pathways linking hormonal milieu, emotional attitudes and cognitive function [6–8]. Stressful conditions activate the hypothalamic–pituitary–adrenal axis with an increase in the secretion of corticotropin-releasing hormone (CRH), adrenocorticotropin (ACTH), cortisol, and endogenous opioids. Endogenous opioids play a significant role in the inhibition of the pulsatile release of both GnRH and LH driven by stress [5]. Numerous other hormones and neurotransmitters, including prolactin, dopamine, neuropeptide Y (NPY), GABA, and serotonin negatively affect GnRH release [5]. Reduction of leptin, a 16 kDa adipokine produced by the adipose tissue, also contributes to the suppression of pulsatile GnRH release in patients with FHA [9]. However, GnRH neurons do not express leptin receptors and therefore other peptides, including kisspeptin, which is the most potent stimulator of GnRH secretion, mediate leptin regulation of hypothalamic GnRH release [10]. Kisspeptin acts directly on hypothalamic GnRH neurons via the kisspeptin receptor and causes GnRH release into the hypophyseal-portal blood circulation. Kisspeptin neurons co-express neurokinin B and dynorphin (KNDy neurons), which act autosynaptically to synchronize the pulsatile secretion of kisspeptin with excitatory (neurokinin B) and inhibitory signals (dynorphin A). Kisspeptin plays a main role in the regulation of reproduction but also of sexual function and other aspects of reproductive behavior from rodents to humans [11]. Hypoestrogenism, the final hormonal consequence of the impairment in GnRH and gonadotropin pulsatile release, may inflect central and peripheral components of sexual function [12]. Hypoandrogenism that becomes evident in severe forms of hypothalamic amenorrhea (HA) [13] may also play a role. Dundon and colleagues [14] reported that women with FHA had more sexual problems than healthy controls. Moreover, psychologic symptoms that contributed to the onset of FHA [7] partially mediated the relationship between FHA and sexual functioning [14].

FHA may coexist with polycystic ovary syndrome (PCOS) [2], another very common cause of hormonal infertility in women of reproductive age, affecting up to 15% worldwide, depending on the diagnostic criteria [15]. Wang and Lobo [16] explored the spectrum of FHA and polycystic ovarian morphology (PCOM) and identified women with FHA and a PCOS-phenotype that may have inherently hyperandrogenic ovaries quiescent because of low gonadotropin secretion resulting from hypothalamic functional suppression. Recent considerations suggest a better distinction between FHA and PCOS is relevant to improve treatment strategies for anovulatory infertility [17]. Indeed, a correct assessment has an important significance both in terms of disease severity and individualized care of FHA based on the hormonal milieu and increased follicle number per ovary [18] without the typical peripheral follicular distribution, the so-called multicystic ovarian morphology (MCOM) [19]. In spite of a real consensus on the ultrasound criteria to define women with FHA carrying PCOS-like ovaries [18, 20], it seems likely they display a peculiar hormonal profile with an elevation of some androgen levels [21–23]. These hormonal features may affect individual well-being, including mood and sexual functioning and define different phenotypes of women with FHA.

That being so, the present retrospective study aimed to investigate sexual function and distress in women with FHA as compared to women with FHA + PCOS-phenotype. Psychometric variables were also measured to look over emotional milieu of these two groups of patients. In addition, we explored the relationship between sexual function and distress with the hormonal profile of FHA women.

## Methods

This is a retrospective study conducted on women referred to the Unit of Gynecological Endocrinology at IRCCS San Matteo Foundation of the University of Pavia, in northwest Italy, for diagnosis and treatment of menstrual dysfunction. The local ethics committee granted approval for the analysis of medical records of those patients who have signed an informed consent.

The patient cohort included women (*n* = 126) with a diagnosis of secondary amenorrhea (hypogonadotropic and normogonadotropic) of hypothalamic origin, according to our standard of practice, over the last 5 years. All women underwent a clinical interview, including a primary care assessment of mental disorders, a physical and gynecological examination, and an ultrasound evaluation. Following the first consultation, each amenorrheic woman underwent a fasting blood sample to measure baseline hormonal profile and appropriate psychometric tests.

The following inclusion criteria were established: age ≥ 16 years (at least 2 years since menarche), body mass index (BMI) ≥ 16 kg/m^2^, the disappearance of menses for at least 3 months before clinical evaluation, low or normal LH levels, 17ß-estradiol (E_2_) levels ≤ 100 pg/ml, and one or more recent predisposing factors (weight loss, excessive physical activity, stressful situations).

The following exclusion criteria were used: pregnancy and lactation, any hormonal therapy (including combined hormonal contraceptives), history of major diseases including endocrine and psychiatric disorders, psychoactive medications, tobacco or illicit drug use, alcohol dependence, overt eating and sleeping disorders.

To serve the scope of investigating sexual functioning, we further excluded those women who did not report a sexual debut and declared not being in a stable sexual relationship in the preceding three months. Finally, we included 79 women with FHA in the present analysis.

According to the Endocrine Society Clinical Practice guideline for the diagnosis and treatment of FHA [2], we reconsidered the records of our study sample to identify a potential PCOS-phenotype, based on menstrual history, clinical signs, and two-dimensional transvaginal ultrasound evaluation. Those FHA women with MCOM, as previously described (six or more small follicles in one plane distributed within normal stroma) [19], were classified as FHA + PCOS-phenotype (*n* = 43), whereas the remaining women as typical FHA (*n* = 36). Endometrial thickness was lower than 4 mm in every woman.

Information about age, age at menarche, education and body mass index (kg/m^2^) were included in the present analysis. Bodyweight (kg) and height (m) were measured in a fasting state, without shoes and without wearing heavy clothes, by using the same calibrated scale and stadiometer. We considered available hormones routinely measured in women with FHA at the first consultation. Assays validated as standard practice in our University Research Hospital were the following: follicle-stimulating hormone (FSH) [follicular phase range 2.8–11.3 IU/L], luteinizing hormone (LH) [follicular phase range 1.1–11.6 IU/L], 17ß-estradiol (E_2_) [follicular phase range 15–160 pg/ml], prolactin [normal range 1.9–25 ng/ml], thyroid-stimulating hormone (TSH) [normal range 0.4–4.0 mIU/L), free triiodothyronine (FT3) [normal range 1.8–4.2 pg/ml]*,* free thyroxine (FT4) [normal range 8.0–19.0 pg/ml], androstenedione [normal range 0.46–3.39 ng/ml], total testosterone (TT) [normal range: 0.15–0.80 ng/ml], dehydroepiandrosterone sulfate (DHEAS) [normal range 0.35–4.3 mcg/ml], cortisol [normal range AM 4.3–22.4 mcg/dl], and insulin [normal range 3–25 mIU/L]. FSH, LH, E2, prolactin, androstenedione, Total T, DHEAS, cortisol were measured by chemiluminescence (SIEMENS Immulite 2000, Germany). The minimal detectable limit for each hormone was as follows: 0.1 IU/L (FSH and LH), 15 pg/ml (E2), 0.5 ng/ml (prolactin), 0.3 ng/ml (androstenedione), 0.15 ng/ml (TT), 0.03 mcg/ml (DHEAS), and 0.2 mcg/dl (cortisol). The inter-assay coefficients of variation were 4.4% (FSH), 6.1% (LH), 7.3% (E2), 5.8% (prolactin), 6.2% (androstenedione), 6.6% (total T), 5.9% (DHEAS), and 6.1% (cortisol). The intra-assay coefficients of variation were 3.8% (FSH), 4.9% (LH), 6.1% (E2), 5.1% (prolactin), 5.5% (androstenedione), 5.5% (total T), 5.1% (DHEAS), and 5.2% (cortisol). Insulin was measured by the ADVIA XPT (Siemens Healthcare; detection limit 0.5/L, inter-assay coefficients of variation 5.9%, intra-assay coefficients of variation 4.6%). TSH (third generation), FT4 and FT3 assays were performed on a fully automated ADVIA Centaur analyzer (Siemens Healthcare Diagnostics) with a sensitivity of 0.02 mIU/L (TSH) and 1.1 pg/ml (FT3, FT4), an inter-assay coefficients of variation of 4.9% (TSH), 4.2% (FT3) and 4.4% (FT4) and an intra-assay coefficients of variation of 3.9% (TSH), 3.6% (FT3) and 3.8% (FT4).

Information deriving from validated psychometric questionnaires to evaluate sexual functioning, as well as other typical intra-personal characteristics of FHA (body image, bulimic risk, mood, and personality), were available and listed as follows:

1. *The female sexual functional index (FSFI),* a 19-item questionnaire developed as a brief, multidimensional self-report instrument for assessing the key domains of sexual function (desire, arousal, lubrication, orgasm, satisfaction, and pain). A score of 0 (1 for desire and satisfaction domains) to 5 is assigned for each response. Scores for each domain and the total score (range 2–36) are provided. The optimal cut-off score for differentiating women between 18 and 74 years with and without sexual problems corresponds to ≤ 26.55 [24].
2. *The female sexual distress scale-revised (FSDS-R),* a 13-item instrument used to measure sexually related personal distress in women with sexual symptoms, including hypoactive sexual desire disorder (HSDD). This questionnaire is based on a 5-point scale (from 0 to 4) with a total score from 0 to 52. The optimal cut-off score for differentiating women with and without sexual distress is ≥ 11 [25].
3. *The body attitude test (BAT)* designed for the assessment of the individual subjective body experience and attitudes. The BAT is composed of 20 items, which are grouped into four main factors: negative appreciation of body size, lack of familiarity with one’s own body, general body dissatisfaction, and rest factor. Each item is rated on a scale from 0 to 5. The maximum score obtained can be 100 points, with 36 being the cut-off point for distinguishing between patients with eating disorders and the general population [26].
4. *The bulimia investigation test (BITE)* [27] validated to assess bulimic risk. The questionnaire investigates habits of dieting and symptoms and behaviors associated with binge eating. Patients are classified, according to their score, as being at low risk (score < 10), at medium risk (score between 10 and 24), and at high risk (score ≥ 25) of having bulimia.
5. *The state anxiety inventory (STAI)*, a 40-item self-report inventory used to assess two types of anxiety symptoms: state anxiety (i.e., how a person in the current situation responds to perceived threat) and trait anxiety (i.e., the stable tendency to attend, experience, and report negative emotions such as fears, worries, and anxiety across many situations). Interpretation is direct, with higher scores on a 4-point scale indicating greater trait or state anxiety (score range from 20 to 80: ≤ 40 = no anxiety; 41–50 = mild; 51–60 = moderate; and > 60 = severe) [28].
6. *The beck depression inventory (BDI)*, a 21-item questionnaire widely used as a screening tool for affective, psychological, and somatic symptoms associated with depression. The total score (ranging from 0 to 63: < 10 = no depression; 10–18 = mild; 19–29 = moderate; and 30–63 = severe) is derived by summing the individual item scores on a Likert scale ranging from 0 to 3 [29].
7. *The multidimensional perfectionism scale (MPS)*, a widely used scale to measure perfectionism. The MPS includes 35 items grouped in 6 subscales: (1) high personal standards; (2) excessive concern about making mistakes; (3) excessive concern about the quality of personal actions; (4) excessively high parental expectations towards the child; (5) excessive parental criticism and blame towards the child for failing to achieve the high standards decided by the parent; (6) appreciation of order, cleanliness, organization. Higher scores on each of the scales reflect greater levels of perfectionism. A cut-off score > 25 has been identified [30].

### Statistical analysis

The Kolmogorov–Smirnov test was used to assess the distribution of each variable. Results are reported as mean ± standard deviation (SD) or median [interquartile range (IQR)] based on their normal or not normal distribution, respectively. Differences in the distribution of the values of the continuous variables between the two study groups were assessed using the Student *t* test or the Mann–Whitney *U* test for normally and not normally distributed variables, respectively. To assess the influence of BMI on outcomes, we used a two-way analysis of variance (ANOVA) to assess the between-group and within-group differences. Diagnosis (FHA or FAH-PCOS) and BMI (normal weight and underweight) were considered as independent variables (factors A and B, respectively), while the FSFI total score, FSDS-R total score, and hormone values as dependent variables. Furthermore, a multiple regression analysis was performed to investigate the association between hormones and psychometric characteristics within the female sexual functioning in the two study groups. Age, education, weight, and BMI were included in the model as possible confounders. The software SPSS 23.0 for Windows (SPSS Inc., Chicago, USA) was used for data analysis. A *p* value lower than 0.05 was considered statistically significant.

## Results

### Demographic and clinical characteristics

The demographic and clinical characteristics of both groups were reported in Table 1. Age, education, and gynecologic age (difference between chronologic age and age at menarche) did not show any statistically significant difference in the two study groups. Age at menarche was significantly higher in women with typical FHA than in women with FHA + PCOS-phenotype. Body weight and BMI were significantly lower in typical FHA women compared to the FHA + PCOS- phenotype group (*p* < 0.001, for both).

**Table 1:** Demographic and clinical characteristics of women with typical functional hypothalamic amenorrhea (FHA) and women with FHA and an underlying polycystic ovarian syndrome (PCOS)-phenotype

	Typical FHA (*n* = 36) Mean ± SD	FHA + PCOS-phenotype (*n* = 43) Mean ± SD	*p* value
Age (years)	26.4 ± 5.4	24.5 ± 5.3	0.10
Education (years)	14.8 ± 2.3	13.9 ± 3.09	0.09
Age at menarche (years)	12.8 ± 1.3	11.8 ± 1.2	< 0.001
Gynecological age (years)	13.6 ± 4.9	12.7 ± 4.05	0.4
Weight (kg)	50.5 ± 5.1	54.4 ± 5.6	< 0.001
BMI (kg/m^2^)	18.5 ± 1.5	20.5 ± 2.2	< 0.001

### Hormonal profile

Table 2 showed the hormonal profile of the two groups of women with FHA. The levels of LH, E_2_, androstenedione, insulin, FreeT3, and FreeT4 were statistically significantly lower in women with typical FHA compared to women with FHA + PCOS phenotype. On the contrary, statistically significant higher prolactin and DHEAS concentrations were found in the FHA + PCOS-phenotype compared to women with typical FHA. FSH, TT, and cortisol were superimposable in the two groups. See Table 2 for individual significance. Interestingly, being normal weight or underweight did not influence the analysis. Indeed, none of the hormone values were found to differ significantly based on the BMI analysis factor or the Diagnosis*BMI analysis factor (Supplementary Table 1).

**Table 2:** Baseline hormonal profile of women with typical functional hypothalamic amenorrhea (FHA) and women with FHA and an underlying polycystic ovarian syndrome (PCOS)-phenotype

	Typical FHA (*n* = 36) Mean ± SD	FHA + PCOS-phenotype (*n* = 43) Mean ± SD	*p* value
FSH (IU/L)	5.5 ± 2.2	6.3 ± 1.7	0.06
LH (IU/L)	1.9 ± 1.0	5.7 ± 2.2	0.001
PRL (ng/ml)	8.1 ± 5.3	13.9 ± 5.2	0.001
TSH (mIU/L)	1.5 ± 0.6	1.7 ± 0.6	0.1
FreeT4 (ng/dl)	9.9 ± 1.8	10.9 ± 1.4	0.01
FreeT3 (pg/ml)	2.6 ± 0.4	3.1 ± 0.4	0.001
E2 (pg/ml)	23.4 ± 11.0	46.0 ± 18.5	0.001
A (ng/ml)	2.1 ± 1.0	2.7 ± 0.8	0.005
T (ng/ml)	0.7 ± 0.4	0.8 ± 0.3	0.25
DHEAS (ng/ml)	1.7 ± 0.7	2.3 ± 0.9	0.002
Cortisol (mcg/dl)	16.6 ± 5.3	15.8 ± 5.7	0.50
Insulin (mIU/ml)	4.1 ± 1.9	7.4 ± 2.1	0.001

### Sexual functioning outcomes

Women with typical FHA reported significantly lower FSFI total score compared to women with FHA + PCOS-phenotype (95% CI for median 16–21.3 vs 21.1–24.1, *p* = 0.002), whereas the FSDS-R score was similar in the two groups (95% CI for median 6–16 vs 6–16.3).

**Fig. 1:**
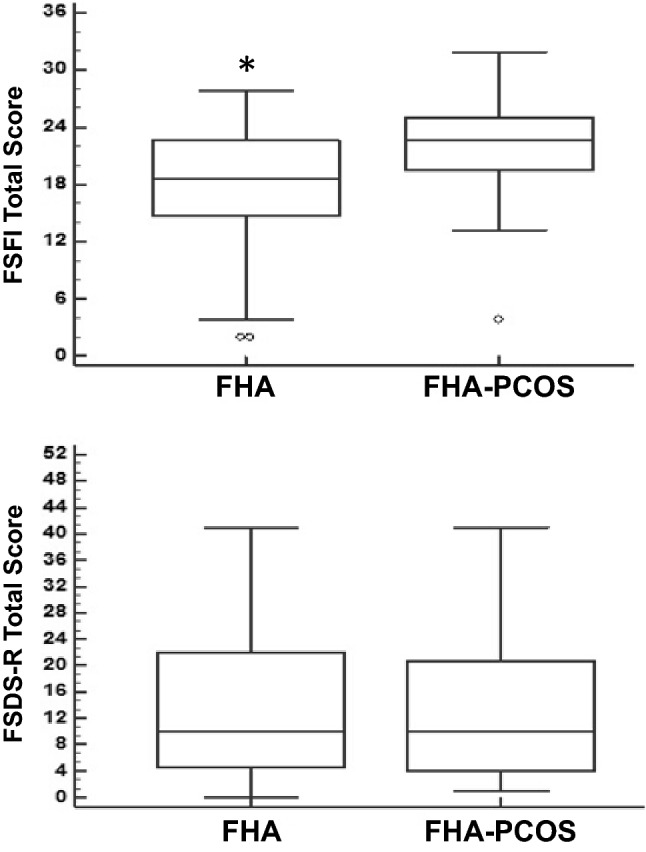
The Female Sexual Function Index (FSFI) total score (top) and the Female Sexual Distress Scale-Revised (FSDS-R) score (bottom) in women with typical functional hypothalamic amenorrhea (FHA) and women with FHA and an underlying polycystic ovarian syndrome (PCOS)-phenotype

Figure 1 showed the median FSFI total score and the median FSDS-R total score of the two groups. The rate of reporting sexual symptoms (FSFI total score ≤ 26.55) (21) was very high and almost superimposable in both groups of women (typical FHA: *n* = 34/36; 94.4% versus FHA + PCOS-phenotype: *n* = 39/43; 90.7%). However, when we considered only those women scoring ≤ 17.25, which corresponded to the lower quartile (LQ < 25%) of the total FSFI score distribution in our entire study sample [31], a significantly higher number (*p* = 0.003) of women with typical FHA (*n* = 15/36; 41.7%) reported a clinically relevant diagnosis compared to women with FHA + PCOS phenotype (*n* = 5/43; 11.6%). The weight variable (normal or underweight) did not appear to influence the analysis, with no difference in the two FSFI scores, based on the BMI analysis factor or the Diagnosis*BMI analysis factor (Supplementary Table 1).

There was no statistically significant difference in the sexual distress reporting rate between the typical FHA phenotype (*n* = 14/36; 38.9%) and FHA + PCOS phenotype (*n* = 18/43; 41.9%). The FSDS-R scores, which may suspect the presence of HSDD, were also similar in the two groups (typical FHA: *n* = 7/36; 19.4% versus FHA + PCOS phenotype: 7/43; 16.3%).

Comparisons for the domains of FSFI (Table 3) outlined women with typical FHA reporting significantly lower scores for desire, arousal, lubrication, and orgasm, but not for sexual satisfaction, compared to women with FHA + PCOS-phenotype. Sexual pain was highly reported in both groups, with a high rate of inter-individual variability in women with FHA + PCOS-phenotype (see Table 3 for individual significance).

**Table 3:** Comparison of Female Sexual Function Index (FSFI) subdomains in women with typical functional hypothalamic amenorrhea (FHA) and women with FHA and an underlying polycystic ovarian syndrome (PCOS)-phenotype

	Typical FHA (*n* = 36) Median (95% CI)	FHA + PCOS-phenotype (*n* = 43) Median (95% CI)	*p* value
Desire	3.3 (2.8–3.6)	3.6 (3.3–4.4)	0.02
Arousal	3.6 (3.0–4.8)	4.5 (3.9–5.1)	0.01
Lubrication	3.1 (2.9–3.3)	3.3 (3.3–3.6)	0.02
Orgasm	3.2 (2.7–4.0)	3.6 (3.4–4.0)	0.03
Satisfaction	4.2 (2.8–4.2)	4.8 (4.2–4.8)	0.08
Pain	1.2 (1.2–1.6)	2.0 (1.2–2.4)	0.27

### Non-sexual function psychometric outcomes

Table 4 showed the comparison of psychometric characteristics in women with standard FHA and those with FHA + PCOS-phenotype. The two groups were very similar with no statistically significant difference in body attitude, state and trait anxiety, depression, bulimic risk, and perfectionism.

**Table 4:** Comparison of some psychometric characteristics in women with typical functional hypothalamic amenorrhea (FHA) and women with FHA and an underlying polycystic ovarian syndrome (PCOS)-phenotype

	Typical FHA (*n* = 36) Median (95% CI)	FHA + PCOS-phenotype (*n* = 43) Median (95% CI)	*p* value
Body attitude (BAT)	32.5 (23.0–36.3)	32 (28.6–34.4)	0.70
State anxiety (STAI-I)	44.0 (39.8–51.0)	42.0 (36.0–50.3)	0.35
Trait anxiety (STAI-2)	47.0 (42.0–50.0)	47.5 (41.0–51.0)	0.99
Depression (BDI)	9.0 (7.0–12.0)	7.5 (5.0–12.0)	0.72
Bulimia (BITE)	8.0 (7.0–12.4)	9.0 (6.7–14.0)	0.65
Perfectionism (MPS)	23.0 (19.0–26.4)	24.5 (21.6–28.3)	0.17

### Correlation of hormonal profile and psychometric characteristics with sexual functioning

**Fig. 2:**
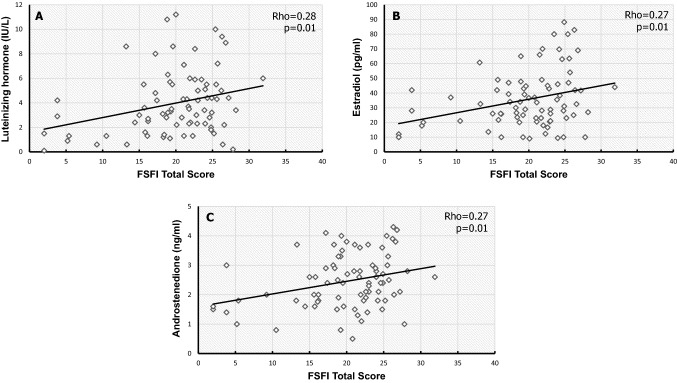
Scatterplots of the correlations between Female Sexual Function Index (FSFI) total score and luteinizing hormone (**A**), estradiol (**B**), and androstenedione (**C**)

When we attempted to correlate the hormonal profile with FSFI total score in typical FHA and FHA + PCOS-phenotype groups, respectively, results were not statistically significant. Interestingly, a positive correlation between LH, androstenedione and E_2_ with total FSFI score was found in the entire FHA population (Fig. 2). Those FHA women with an FSFI total score in the LQ of distribution (LQ = 17.25, *n* = 20) showed significantly lower LH (2.4 IU/L, 95% CI 1.3–3.5.6) and androstenedione (1.8 ng/ml, 95% CI 1.6–2.5) levels compared to the remaining group (LH 4.2 UI/L, 95% CI 3.2–4.7 and androstenedione 2.6 ng/ml, 95% CI 2.3–2.8; *p* = 0.01 and *p* = 0.02, respectively).

State anxiety was negatively correlated with total FSFI score in both women with typical FHA (rho: − 0.33, *p* = 0.05) and FHA + PCOS-phenotype (rho: − 0.40, *p* = 0.009). A significant negative correlation was also found between the total FSFI score and trait anxiety and depression (rho: − 0.35, *p* = 0.02 and rho: − 0.46, *p* = 0.002, respectively) only in the FHA + PCOS-phenotype.

Supplementary Tables S2–S5 reported correlations between FSFI domains, hormonal profile, and psychometric characteristics in both women with typical FHA and FHA + PCOS-phenotype.

## Discussion

The present study demonstrated that women with FHA have a very high rate of sexual symptoms as part of their menstrual dysfunction, especially those with typical FHA as compared to FHA-PCOS-phenotype. Sexual distress was present to a lower extent in both groups, without any significant difference, in line with similar levels of sexual satisfaction. Interestingly, a potential role of sex hormones, mainly LH-driven androstenedione, in influencing some components of women’s sexual response (desire, arousal, lubrication, and orgasm) emerged, with high sexual pain scores in both FHA groups, regardless of circulating E_2_ or androgen levels.

Typical FHA and FHA + PCOS phenotypes were superimposable from a psychosocial standpoint evaluated by body attitude, state and trait anxiety, depression, bulimic risk, and perfectionism. Our psychometric data were consistent with the neuropsychological profile of FHA adolescent [32] and young adult [7] women reported in previous studies and confirmed that typical FHA and FHA + PCOS-phenotype represent a continuum on a spectrum of menstrual dysfunction. That being so, the peculiar psychoneuroendocrine environment of women with FHA [33] is likely to play a fundamental role in sexual functioning in both groups. However, our attempt to compare typical FHA with FHA + PCOS-phenotype outlined a greater impairment in sexual function of those women with more profound hypogonadism. Whether abnormalities of sex hormones may significantly contribute to psychosexual well-being of women with menstrual dysfunction awaits further evidence in prospective studies with sensitive assays. In a cross-sectional study, circulating androgens (total and free testosterone, androstenedione, and DHEAS), measured by mass spectrometry, correlated to the FSFI sexual desire domain score, especially in a subgroup of women aged 25–44 years [34]. Given the multidimensionality of sexual functioning, controversies in linking levels of androgens to specific domains of the sexual response still exist in many gynecological conditions, including PCOS [35]. A recent meta-analysis including 34 studies and 3268 women with a mean age of 36.5 years showed that total testosterone was associated with better sexual function [SMD = 0.55 (0.28;0.82), *p* < 0.0001] [36]. FHA is among the potential causes of low testosterone due to anovulation, possibly leading to HSDD [37]. Moreover, both hypoestrogenism [38] and hypoandrogenism [39] may contribute to the clinical manifestation of sexual symptoms associated with genital involution and poor responsiveness. However, two subsequent consensus publications of the International Society of Sexual Medicine (ISSM) recognized the paucity of research on the potential impact of FHA and its treatment on sexual functioning [40, 41]. An early study [42] explored the possibility that impaired sexual function may result from reduced levels of testosterone in subjects with secondary amenorrhea of hypothalamic origin diagnosed according to the presence of risk factors (i.e., weight loss before the onset of amenorrhea, low body weight, strenuous exercise, and vegetarianism). Eight women with FHA associated with these particular life-styles demonstrated impaired sexual function and significantly lower circulating testosterone levels as compared with 8 normally menstruating women [42]. Interestingly, women with FHA were asked to produce erotic fantasies demonstrating a reduced capacity for sexual fantasizing, less subjective sexual excitement, and less vaginal vasocongestion as compared to healthy women. Treatment with testosterone undecanoate (40 mg) increased vaginal vasocongestion in women with FHA without affecting the subjective sexual experience in response to the exposure to an erotic movie [42].

Sex hormone-binding globulin (SHBG) has been previously reported as a promising parameter to distinguish between FHA and PCOS [18]. Indeed, SHBG levels were lower in PCOS patients, regardless of BMI [18]. Moreover, SHBG has been related to sexual function because oligomenorrheic and/or hirsute women with values lower than 33.4 nmol/l displayed adverse metabolic profiles along with higher clitoral pulsatility index (PI), an index of vascular resistance [43]. These results agreed with recent literature reporting a higher clitoral PI in patients with metabolic impairment and sexual dysfunction [44–46]. Results from a substudy of the Grollo-Ruzzene cross-sectional study, which recruited 761 premenopausal women from eastern states in Australia, showed a negative association between sexual desire and SHBG, in line with other studies conducted on premenopausal women [47]. On the other hand, a recent cross-sectional study showed a positive correlation between SHBG and total FSFI score (*r* = 0.39; *p* = 0.02) in 36 postmenopausal women (age range 45–65 years) [48]. Of note, SHBG was positively correlated with three specific FSFI domains [orgasm (*r* = 0.39; *p* = 0.01), satisfaction (*r* = 0.33; *p* = 0.04), and pain (*r* = 0.44; *p* < 0.01)]. Collectively, these findings suggest the role of SHBG in modulating female sexual function merits further investigation. Unfortunately, SHBG data were not available in our present study. Among factors that can influence SHBG levels, thyroid hormones played a significant role. In our sample, levels of FreeT3 and FreeT4 were statistically significantly lower in women with typical FHA compared to women with FHA + PCOS phenotype. It is a matter of fact that both hypothyroidism and hyperthyroidism are associated with changes in concentrations of SHBG, testosterone, and E_2_. However, the direct relationship between thyroid function and sexual function is still not clear. Indeed, a recent study reported that, although genetically predicted thyroid function was associated with sex hormones, it was not associated with ovulatory function in women (duration of menstrual cycle, age at menarche and menopause and reproductive lifespan) and erectile dysfunction in men [49].

In our study sample, less than half of women with FHA was clinically distressed by sexual symptoms and only a few of them reported low sexual desire associated with sexual distress (HSDD). These data supported previous evidence of similar satisfaction with a sexual relationship with respect to healthy women [14]. Sexual symptoms were confirmed to co-occur in FHA women more vulnerable to mood disorders [14] as it was found in general clinical practice [50]. Even in a nonclinical sample of young women, depression-specific anhedonia and sexual desire were closely related [51]. Moreover, in the same study physiological hyperarousal linked to anxiety gave rise to sexual arousal difficulties and vaginal pain [51]. In women with FHA, the common link might be the increased activity of hypothalamus–pituitary–adrenal axis (HPA) associated with the impairment of some central pathways (opioids and serotonin) [52], which play a fundamental role in the neuroendocrine balance driving sexual functioning [6] and also contribute to the onset of menstrual dysfunction [4]. On the other hand, other common pathways connecting the areas of emotion dysregulation/impulsivity and perfectionism/over-control both with eating disorder-specific psychopathology and with different types of sexual impairment [53] might explain the high rate of sexual symptoms in women with FHA, including sexual pain, reported in the present study.

The major limitations of our study are the retrospective design and the lack of sensitive assays to measure androgens. On the other hand, the present study has the strength that it was conducted by using validated instruments encompassing several psychosexual dimensions in a clinically and hormonally well-characterized sample of women with FHA. Being aware of the paucity of data investigating sexual functioning in amenorrheic women, we aimed to present real-life data to guide the daily practice of every clinician dealing with anovulatory menstrual disorders. Whether the experience of infertility associated with the absence of menstrual periodicity contributes to the manifestation of sexual symptoms is presently unknown and it awaits further investigation.

Overall, our findings underscored the need to include sexual counselling in the diagnostic evaluation for FHA. In addition, appropriate management should provide not only cognitive-behavioral therapy [54] to overcome risk factors, such as mood disorders, dysfunctional attitudes, eating disorders, body image issues, excessive exercise, and other stressors, but also sexual symptoms. Tailored hormonal strategies should be investigated also in the context of sexual care.

## Conclusions

In conclusion, FHA is a suitable paradigm to investigate the complexity of sexual functioning from both a neuroendocrine and a psychosocial standpoint. Along with many other stressors, we cannot exclude that a high rate of sexual symptoms may contribute to the maintenance of such menstrual dysfunction. Moreover, the recognition of an underlying PCOS-phenotype can be helpful to investigate discrete components of sexual functioning possibly linked to different profiles of hypogonadism. Prospective studies are warranted to determine the relevance of our retrospective data in the clinical management of women with FHA.

## Supplementary Information

Below is the link to the electronic supplementary material.

Supplementary file1 (DOCX 28 KB)

## Data Availability

All data generated or analysed during this study are included in this published article and its supplementary information files.
