# Supplementary material for: Sexual functioning in women with functional hypothalamic amenorrhea: exploring the relevance of an underlying polycystic ovary syndrome (PCOS)-phenotype
Source: J Endocrinol Invest. 2023 Feb 3;46(8):1623–32. doi: 10.1007/s40618-023-02021-7 (PMC10348938; doi:10.1007/s40618-023-02021-7)
Supplement: Supplementary file 1 — Supplementary file1 (DOCX 28 KB) [file 40618_2023_2021_MOESM1_ESM.docx]

**Supplementary Tables**

**Supplementary Table 1.** Test of between-subjects effects

|  | **Sum of Squares** | **DF** | **Mean Square** | **F** | **P** |
| --- | --- | --- | --- | --- | --- |
| **FSFI Total Score** |  |  |  |  |  |
| ***Diagnosis (FHA/FHA-PCOS)*** | 280.672 | 1 | 280.672 | 8.198 | 0.005 |
| ***BMI (Normal weight – Underweight)*** | 67.849 | 1 | 67.849 | 1.982 | 0.163 |
| ***Diagnosis*BMI*** | 14.460 | 1 | 14.460 | 0.422 | 0 |
| ***Residuals*** | 2567.715 | 75 | 34.236 |  |  |
| **FSFI Total Score** |  |  |  |  |  |
| ***Diagnosis (FHA/FHA-PCOS)*** | 28.600 | 1 | 28.600 | 0.217 | 0.643 |
| ***BMI (Normal weight – Underweight)*** | 80.373 | 1 | 80.373 | 0.609 | 0.438 |
| ***Diagnosis*BMI*** | 62.515 | 1 | 62.515 | 0.474 | 0.494 |
| ***Residuals*** | 9902.109 | 75 | 132.028 |  |  |
| ***FSH (IU/L)*** |  |  |  |  |  |
| ***Diagnosis (FHA/FHA-PCOS)*** | 6.699 | 1 | 6.699 | 1.790 | 0.185 |
| ***BMI (Normal weight – Underweight)*** | 1.531 | 1 | 1.531 | 0.409 | 0.524 |
| ***Diagnosis*BMI*** | 10.133 | 1 | 10.133 | 2.708 | 0.104 |
| ***Residuals*** | 280.620 | 75 | 3.742 |  |  |
| ***LH (IU/L)*** |  |  |  |  |  |
| ***Diagnosis (FHA/FHA-PCOS)*** | 183.216 | 1 | 183.216 | 59.187 | <0.001 |
| ***BMI (Normal weight – Underweight)*** | 1.600 | 1 | 1.600 | 0.517 | 0.474 |
| ***Diagnosis*BMI*** | 5.533 | 1 | 5.533 | 1.787 | 0.185 |
| ***Residuals*** | 232.167 | 75 | 3.096 |  |  |
| ***PRL (ng/ml)*** |  |  |  |  |  |
| ***Diagnosis (FHA/FHA-PCOS)*** | 465.003 | 1 | 465.003 | 18.141 | <0.001 |
| ***BMI (Normal weight – Underweight)*** | 75.562 | 1 | 75.562 | 2.948 | 0.090 |
| ***Diagnosis*BMI*** | 99.439 | 1 | 99.439 | 3.879 | 0.053 |
| ***Residuals*** | 1922.487 | 75 | 25.633 |  |  |
| ***TSH (mIU/L)*** |  |  |  |  |  |
| ***Diagnosis (FHA/FHA-PCOS)*** | 1.579 | 1 | 1.579 | 4.628 | 0.035 |
| ***BMI (Normal weight – Underweight)*** | 0.566 | 1 | 0.566 | 1.659 | 0.202 |
| ***Diagnosis*BMI*** | 0.313 | 1 | 0.313 | 0.916 | 0.342 |
| ***Residuals*** | 25.586 | 75 | 0.341 |  |  |
| ***FreeT4 (ng/dl)*** |  |  |  |  |  |
| ***Diagnosis (FHA/FHA-PCOS)*** | 20.147 | 1 | 20.147 | 7.991 | 0.006 |
| ***BMI (Normal weight – Underweight)*** | 0.0114 | 1 | 0.0114 | 0.00454 | 0.946 |
| ***Diagnosis*BMI*** | 6.244 | 1 | 6.244 | 2.477 | 0.120 |
| ***Residuals*** | 189.079 | 75 | 2.521 |  |  |
| ***FreeT3 (pg/ml)*** |  |  |  |  |  |
| ***Diagnosis (FHA/FHA-PCOS)*** | 4.194 | 1 | 4.194 | 24.888 | <0.001 |
| ***BMI (Normal weight – Underweight)*** | 0.0252 | 1 | 0.0252 | 0.450 | 0.700 |
| ***Diagnosis*BMI*** | 0.0944 | 1 | 0.0944 | 0.560 | 0.457 |
| ***Residuals*** | 12.640 | 75 | 0.169 |  |  |
| ***E2 (pg/ml)*** |  |  |  |  |  |
| ***Diagnosis (FHA/FHA-PCOS)*** | 8403.834 | 1 | 8403.834 | 34.030 | <0.001 |
| ***BMI (Normal weight – Underweight)*** | 72.693 | 1 | 72.693 | 0.294 | 0.589 |
| ***Diagnosis*BMI*** | 14.857 | 1 | 14.857 | 0.0602 | 0.807 |
| ***Residuals*** | 18521.478 | 75 | 246.953 |  |  |
| ***A (ng/ml)*** |  |  |  |  |  |
| ***Diagnosis (FHA/FHA-PCOS)*** | 3.553 | 1 | 3.553 | 4.589 | 0.035 |
| ***BMI (Normal weight – Underweight)*** | 0.0914 | 1 | 0.0914 | 0.118 | 0.732 |
| ***Diagnosis*BMI*** | 0.485 | 1 | 0.485 | 0.627 | 0.431 |
| ***Residuals*** | 58.070 | 75 | 0.774 |  |  |
| ***T (ng/ml)*** |  |  |  |  |  |
| ***Diagnosis (FHA/FHA-PCOS)*** | 0.0754 | 1 | 0.0754 | 0.595 | 0.443 |
| ***BMI (Normal weight – Underweight)*** | 0.0552 | 1 | 0.0552 | 0.436 | 0.511 |
| ***Diagnosis*BMI*** | 0.00151 | 1 | 0.00151 | 0.0119 | 0.913 |
| ***Residuals*** | 9.496 | 75 | 0.127 |  |  |
| ***DHEAS (ng/ml)*** |  |  |  |  |  |
| ***Diagnosis (FHA/FHA-PCOS)*** | 17.807 | 1 | 17.807 | 2.436 | 0.123 |
| ***BMI (Normal weight – Underweight)*** | 1.365 | 1 | 1.365 | 0.187 | 0.667 |
| ***Diagnosis*BMI*** | 1.101 | 1 | 1.101 | 0.151 | 0.699 |
| ***Residuals*** | 548.281 | 75 | 7.310 |  |  |
| ***Cortisol (mcg/dl)*** |  |  |  |  |  |
| ***Diagnosis (FHA/FHA-PCOS)*** | 1.606 | 1 | 1.606 | 0.0516 | 0.821 |
| ***BMI (Normal weight – Underweight)*** | 23.976 | 1 | 23.976 | 0.770 | 0.383 |
| ***Diagnosis*BMI*** | 3.012 | 1 | 3.012 | 0.0967 | 0.757 |
| ***Residuals*** | 2335.526 | 75 | 31.140 |  |  |
| ***Insulin (mIU/ml)*** |  |  |  |  |  |
| ***Diagnosis (FHA/FHA-PCOS)*** | 136.958 | 1 | 136.985 | 33.865 | <0.001 |
| ***BMI (Normal weight – Underweight)*** | 0.000118 | 1 | 0.000118 | 0.0000292 | 0.996 |
| ***Diagnosis*BMI*** | 9.878 | 1 | 9.878 | 2.442 | 0.122 |
| ***Residuals*** | 303.374 | 75 | 4.045 |  |  |

*Legend:* FSFI-T: Female Sexual Function Index-total score; FSH: follicle-stimulating hormone; LH:  luteinizing hormone; PRL: prolactin; TSH: Thyroid stimulating hormone; A: androstenedione; DHEAS: Dehydroepiandrosterone sulfate; T: testosterone; FT3: free triiodothyronine; FT4: free thyroxine; E2: Estradiol.

**Table 2. Spearman rank correlation analyses between hormonal profile and sexual functioning in typical FHA women (n=36).**

|  | **FSH** | **LH** | **E2** | **PRL** | **A** | **DHEAS** | **T** | **INSULIN** | **TSH** | **FT3** | **FT4** | **CORTISOL** |
| --- | --- | --- | --- | --- | --- | --- | --- | --- | --- | --- | --- | --- |
| **FSFI-D** | Rho:0.06; p=0.7 | Rho:0.07; p=0.7 | Rho:-0.65; p=0.7 | Rho:-0.06; p=0.7 | Rho:-0.27; p=0.1 | Rho:-0.14; p=0.9 | Rho:-0.09; p=0.6 | Rho:0.11; p=0.5 | Rho:0.02; p=0.9 | Rho:-0.15; p=0.4 | Rho:0.11; p=0.5 | **Rho:-0.34; p=0.04*** |
| **FSFI-A** | Rho:0.14; p=0.4 | Rho:0.18; p=0.3 | Rho:-0.17; p=0.9 | Rho:0.17; p=0.3 | Rho:0.06; p=0.7 | Rho:-0.01; p=0.9 | Rho:0.07; p=0.7 | Rho:0.29; p=0.09 | Rho:0.24; p=0.9 | Rho:0.18; p=0.3 | Rho:-0.26; p=0.1 | **Rho:-0.34; p=0.04*** |
| **FSFI-L** | Rho:0.25; p=0.15 | **Rho:0.33; p=0.04*** | Rho:0.16; p=0.4 | Rho:0.27; p=0.1 | Rho:0.26; p=0.1 | Rho:0.09; p=0.6 | Rho:-0.03; p=0.9 | Rho:0.13; p=0.4 | Rho:-0.17; p=0.9 | Rho:-0.05; p=0.8 | Rho:0.008; p=0.9 | Rho:-0.10; p=0.5 |
| **FSFI-O** | Rho:0.11; p=0.5 | Rho:0.27; p=0.1 | Rho:0.16; p=0.4 | Rho:0.08; p=0.7 | Rho:0.02; p=0.9 | Rho:-0.3; p=0.9 | Rho:0.05; p=0.8 | Rho:0.03; p=0.9 | Rho:-0.06; p=0.7 | Rho:0.02; p=0.9 | Rho:- 0.04; p=0.8 | **Rho:- 0.3; p=0.04*** |
| **FSFI-S** | Rho:0.12; p=0.5 | Rho:-0.07; p=0.7 | Rho:-0.19; p=0.3 | Rho:0.09; p=0.6 | Rho:0.23; p=0.2 | **Rho:0.36; p=0.04*** | Rho:0.18; p=0.3 | Rho:0.2; p=0.2 | Rho:-0.02; p=0.9 | Rho:-0.002; p=0.9 | Rho:-0.07; p=0.7 | Rho:-0.17; p=0.3 |
| **FSFI-P** | Rho:-0.07; p=0.7 | Rho:0.03; p=0.9 | Rho:0.28; p=0.1 | Rho:0.10; p=0.6 | Rho:0.11; p=0.5 | Rho:-0.16; p=0.4 | Rho:0.16; p=0.4 | Rho:-0.32; p=0.6 | Rho:-0.007; p=0.9 | Rho:-0.22; p=0.2 | **Rho:0.39; p=0.02*** | Rho:0.23; p=0.2 |
| **FSFI-T** | Rho:0.12; p=0.48 | Rho:0.09; p=0.6 | Rho:-0.001; p=0.9 | Rho:0.11; p=0.5 | Rho:0.09; p=0.6 | Rho:0.11; p=0.5 | Rho:0.16; p=0.4 | Rho:0.09; p=0.6 | Rho:-0.001; p=0.9 | Rho:-0.12; p=0.5 | Rho:-0.13; p=0.9 | Rho:-0.23; p=0.2 |

*Legend:* FSFI-D: Female Sexual Function Index-Desire; FSFI-A: Female Sexual Function Index-Lubrication; FSFI-O=Female Sexual Function Index-Orgasm; FSFI-S: Female Sexual Function Index-Satisfaction; FSFI-P: Female Sexual Function Index-Pain; FSFI-T: FSFI-total score; FSH: follicle-stimulating hormone; LH:  luteinizing hormone; PRL: prolactin; TSH: Thyroid stimulating hormone; A: androstenedione; DHEAS: Dehydroepiandrosterone sulfate; T: testosterone; FT3: free triiodothyronine; FT4: free thyroxine; E2: Estradiol.

**Table 3. Spearman rank correlation analyses between psychometric characteristics and sexual functioning in typical FHA women (n=36).**

|  | **BAT** | **MPS** | **STAI1** | **STAI2** | **BDI** | **BITE** |
| --- | --- | --- | --- | --- | --- | --- |
| **FSFI-D** | Rho:-0.21; p=0.3 | Rho:-0.16; p=0.4 | Rho:-0.23; p=0.18 | Rho:-0.25; p=0.14 | Rho:-0.44; p=0.8 | Rho:-0.13; p=0.45 |
| **FSFI-A** | Rho:-0.29; p=0.09 | Rho:-0.25; p=0.14 | Rho:-0.20; p=0.2 | Rho:-0.19; p=0.3 | Rho:-0.05; p=0.8 | Rho:-0.12; p=0.5 |
| **FSFI-L** | Rho:-0.09; p=0.6 | Rho:-0.06; p=0.7 | Rho:-0.1; p=0.5 | Rho:-0.03; p=0.9 | Rho:0.02; p=0.9 | Rho:-0.26; p=0.1 |
| **FSFI-O** | Rho:-0.22; p=0.2 | Rho:-0.17; p=0.3 | Rho:-0.24; p=0.2 | Rho:-0.18; p=0.3 | Rho:0.17; p=0.9 | Rho:-0.07; p=0.7 |
| **FSFI-S** | **Rho:-0.39; p=0.02*** | Rho:-0.31; p=0.07 | **Rho:-0.41; p=0.009*** | **Rho:-0.36; p=0.03*** | Rho:-0.23; p=0.18 | Rho:-0.22; p=0.2 |
| **FSFI-P** | Rho:0.24; p=0.2 | Rho:0.13; p=0.5 | Rho:0.07; p=0.7 | Rho:0.07; p=0.7 | Rho:0.27; p=0.1 | Rho:0.12; p=0.5 |
| **FSFI-T** | Rho:-0.26; p=0.1 | Rho:-0.18; p=0.3 | **Rho:-0.33; p=0.05*** | Rho:-0.25; p=0.1 | Rho:-0.01; p=0.9 | Rho:-0.12; p=0.9 |

*Legend:* FSFI-D: Female Sexual Function Index-Desire; FSFI-A: Female Sexual Function Index-Lubrication; FSFI-O=Female Sexual Function Index-Orgasm; FSFI-S: Female Sexual Function Index-Satisfaction; FSFI-P: Female Sexual Function Index-Pain; FSFI-T: FSFI-total score; BAT: Body Attitude Test; BITE: Bulimia Investigation Test; STAI1: State Anxiety Inventory-state anxiety; STAI2: State Anxiety Inventory-trait anxiety; BDI: Beck Depression Inventory; MPS: Multidimensional Perfectionism Scale.

**Table 4. Spearman rank correlation analyses between hormonal profile and sexual functioning in women with FHA+ PCOS-phenotype (n=43).**

|  | **FSH** | **LH** | **E2** | **PRL** | **A** | **DHEAS** | **T** | **INSULIN** | **TSH** | **FT3** | **FT4** | **CORTISOL** |
| --- | --- | --- | --- | --- | --- | --- | --- | --- | --- | --- | --- | --- |
| **FSFI-D** | Rho:-0.68; p=0.7 | Rho:-0.28; p=0.7 | Rho:-0.03; p=0.8 | Rho:-0.11; p=0.5 | Rho:0.21; p=0.2 | Rho:0.29; p=0.06 | Rho:-0.12; p=0.5 | Rho:-0.23; p=0.1 | Rho:-0.12; p=0.5 | Rho:-0.16; p=0.9 | Rho:0.44; p=0.8 | Rho:-0.25; p=0.1 |
| **FSFI-A** | Rho:0.12; p=0.5 | Rho:-0.14; p=0.4 | Rho: 0.13; p=0.4 | Rho:-0.11; p=0.5 | **Rho:0.31; p=0.04*** | Rho:0.05; p=0.8 | Rho:-0.08; p=0.9 | Rho:-0.14; p=0.4 | Rho:-0.32; p=0.8 | Rho:-0.08; p=0.6 | Rho:-0.05; p=0.7 | Rho:-0.13; p=0.4 |
| **FSFI-L** | Rho:0.10; p=0.5 | Rho:0.21; p=0.2 | Rho:0.22; p=0.2 | Rho:0.07; p=0.6 | Rho:-0.05; p=0.8 | Rho:-0.09; p=0.6 | Rho:0.05; p=0.8 | Rho:-0.14; p=0.4 | Rho:-0.05; p=0.8 | Rho:0.04; p=0.8 | Rho:0.001; p=0.9 | Rho:-0.12; p=0.4 |
| **FSFI-O** | Rho:0.09; p=0.5 | Rho:-0.002; p=0.9 | Rho:0.09; p=0.9 | Rho:-0.12; p=0.4 | Rho:0.21; p=0.2 | Rho:-0.25; p=0.1 | Rho:0.20; p=0.2 | Rho:-0.16; p=0.3 | Rho:0.19; p=0.2 | Rho:-0.20; p=0.2 | Rho:-0.16; p=0.3 | Rho:-0.23; p=0.9 |
| **FSFI-S** | **Rho:0.43; p=0.04*** | Rho:0.16; p=0.3 | Rho:0.13; p=0.4 | Rho:-0.04; p=0.8 | Rho:0.11; p=0.5 | Rho:-0.05; p=0.7 | Rho:-0.03; p=0.9 | Rho:-0.04; p=0.8 | Rho:0.007; p=0.9 | Rho:-0.28; p=0.07 | Rho:-0.26; p=0.09 | Rho:-0.19; p=0.2 |
| **FSFI-P** | Rho:0.06; p=0.7 | Rho:0.008; p=0.9 | Rho:0.09; p=0.6 | Rho:-0.18; p=0.2 | Rho:0.04; p=0.8 | Rho:-0.03; p=0.9 | Rho:0.09; p=0.5 | Rho:0.14; p=0.4 | Rho:-0.11; p=0.5 | Rho:0.19; p=0.2 | Rho:0.18; p=0.2 | Rho:0.03; p=0.8 |
| **FSFI-T** | Rho:0.16; p=0.3 | Rho:-0.04; p=0.8 | Rho:0.17; p=0.3 | Rho:-0.14; p=0.4 | Rho:0.24; p=0.1 | Rho:-0.06; p=0.9 | Rho:0.05; p=0.7 | Rho:-0.18; p=0.3 | Rho:0.02; p=0.9 | Rho:-0.11; p=0.5 | Rho:-0.06; p=0.7 | Rho:-0.11; p=0.5 |

*Legend:* FSFI-D: Female Sexual Function Index-Desire; FSFI-A: Female Sexual Function Index-Lubrication; FSFI-O=Female Sexual Function Index-Orgasm; FSFI-S: Female Sexual Function Index-Satisfaction; FSFI-P: Female Sexual Function Index-Pain; FSFI-T: FSFI-total score; FSH: follicle-stimulating hormone; LH:  luteinizing hormone; PRL: prolactin; TSH: Thyroid stimulating hormone; A: androstenedione; DHEAS: Dehydroepiandrosterone sulfate; T: testosterone; FT3: free triiodothyronine; FT4: free thyroxine; E2: Estradiol.

**Table 5. Spearman rank correlation analyses between psychometric characteristics and sexual functioning in women with FHA+ PCOS-phenotype (n=43).**

|  | **BAT** | **MPS** | **STAI1** | **STAI2** | **BDI** | **BITE** |
| --- | --- | --- | --- | --- | --- | --- |
| **FSFI-D** | Rho:0.03; p=0.9 | Rho:0.29; p=0.05 | Rho:-0.28; p=0.07 | Rho:-0.14; p=0.4 | Rho:-0.29; p=0.05 | Rho:0.02; p=0.9 |
| **FSFI-A** | Rho:0.06; p=0.8 | Rho:0.05; p=0.7 | **Rho-:0.34; p=0.02*** | **Rho:-0.31; p=0.04*** | **Rho:-0.42; p=0.005*** | Rho:0.08; p=0.6 |
| **FSFI-L** | Rho:0.24; p=0.1 | Rho:0.28; p=0.07 | Rho:-0.09; p=0.6 | Rho:0.22; p=0.2 | Rho:0.14; p=0.4 | Rho:0.06; p=0.7 |
| **FSFI-O** | Rho:0.02; p=0.9 | Rho:-0.08; p=0.6 | Rho:-0.26; p=0.09 | **Rho:-0.37; p=0.01*** | **Rho:-0.42; p=0.006*** | Rho:-0.09; p=0.6 |
| **FSFI-S** | Rho:-0.19; p=0.2 | Rho:-0.18; p=0.2 | **Rho:-0.5; p=0.001*** | **Rho:-0.48; p=0.001*** | **Rho:-0.43; p=0.004*** | Rho:-0.16; p=0.3 |
| **FSFI-P** | Rho:-0.09; p=0.6 | Rho:0.12; p=0.4 | Rho:0.25; p=0.1 | Rho:0.1; p=0.5 | Rho:0.15; p=0.3 | Rho:-0.06; p=0.7 |
| **FSFI-T** | Rho:-0.05; p=0.8 | Rho:0.12; p=0.4 | **Rho:-0.40; p=0.009*** | **Rho:-0.35; p=0.02*** | **Rho:-0.45; p=0.002*** | Rho:-0.02; p=0.9 |

*Legend:* FSFI-D: Female Sexual Function Index-Desire; FSFI-A: Female Sexual Function Index-Lubrication; FSFI-O=Female Sexual Function Index-Orgasm; FSFI-S: Female Sexual Function Index-Satisfaction; FSFI-P: Female Sexual Function Index-Pain; FSFI-T: FSFI-total score; BAT: Body Attitude Test; BITE: Bulimia Investigation Test; STAI1: State Anxiety Inventory-state anxiety; STAI2: State Anxiety Inventory-trait anxiety; BDI: Beck Depression Inventory; MPS: Multidimensional Perfectionism Scale.
